# An Extracellular Matrix-Based Signature Associated With Immune Microenvironment Predicts the Prognosis and Therapeutic Responses of Patients With Oesophageal Squamous Cell Carcinoma

**DOI:** 10.3389/fmolb.2021.598427

**Published:** 2021-03-18

**Authors:** Hongpan Zhang, Qi Shi, Zhihao Yang, Kaige Wang, Zhiyu Zhang, Zheng Huang, Xiaobin Cui, Feng Li

**Affiliations:** ^1^Department of Pathology and Medical Research Center, Beijing Chaoyang Hospital, Capital Medical University, Beijing, China; ^2^Department of Pathology and Key Laboratory for Xinjiang Endemic and Ethnic Diseases, Shihezi University School of Medicine, Shihezi, China

**Keywords:** ESCC, ECM-related genes, signature, prognosis, therapeutic responses

## Abstract

Evidence has suggested that the cancer-associated extracellular matrix (ECM) could be recognised as immune-related biomarkers that modulate tumour progression and expansion. However, the ECM-associated immune effect on esophageal squamous cell carcinoma (ESCC) prognosis and therapy has not been well characterised. In our study, we first constructed an ECM-related signature including four genes CST1, NELL2, ADAMTSL4, and ANGPTL7 by multivariate Cox regression analyses. This signature could serve as a marker to evaluate the prognosis of patients with ESCC and was successfully validated in testing and combined (training plus testing) cohorts. We also found that there were significant different therapeutic responses to chemotherapy and targeted drugs between the high-risk and low-risk groups of patients defined by the signature. Furthermore, the expression of four genes and immune function analysis suggested that this *ECM-related signature gene might play important roles in the changes of* the tumour microenvironment. In conclusion, our findings demonstrated that the ECM-related signature might serve as an independent prognostic factor and provide a potential biomarker for chemotherapy responses for patients with ESCC.

## Introduction

Esophageal cancer (ESCA), as the seventh most common cancer worldwide, is a widespread human malignancy (Bray et al., 2018). This type of carcinoma includes two main subtypes: esophageal adenocarcinoma and esophageal squamous cell carcinoma (ESCC). ESCC is a common malignancy with a high incidence rate in China (Wei et al., 2015; Deng et al., 2018). Despite advances in surgical techniques and other treatment strategies, the survival of patients with ESCC has not been obviously improved. In recent years, immunotherapy has emerged as a novel treatment strategy and as an effective and promising option for the treatment of various types of cancers (Kono et al., 2018; Mimura et al., 2018; Antoni and Dhabhar, 2019; Fan et al., 2019), including ESCA (Doi et al., 2018; Shah et al., 2019). New clinical trials also indicate that nivolumab significantly improves overall survival compared to chemotherapy and may prove to be a new standard second-line treatment for patients with ESCC (Kato et al., 2019). However, immunotherapy of ESCC remains at an initial stage, exploring the novel immune-related biomarkers is in demand for the evaluation of the prognosis and chemotherapy responses of ESCC.

The tumour microenvironment (TME) consists of an insoluble extracellular matrix (ECM), a stroma composed of fibroblasts, adipocytes, endothelial, and resident immune cells, and a multitude of growth factors and cytokines (Iyengar et al., 2016; Binnewies et al., 2018; DeBerardinis, 2020). The ECM was originally described as the non-cellular component of tissue that provides both biochemical and essential structural support for its cellular constituents, providing functions ranging from cellular adhesion and motility to cell signalling (Hynes, 2009; Theocharis et al., 2016). Accumulating evidence has shown that the ECM conveys specific signals to cells, thereby modulating immune cell migration, activation, proliferation, and differentiation (Pickup et al., 2014; Sangaletti et al., 2017; Mushtaq et al., 2018). Although the cancer-associated ECM plays a very important role in TME, the ECM-associated immune effect on ESCC prognosis and therapy has not been well studied. In this paper, we propose and validate a new ECM-related signature (CST1, ANGPTL7, ADAMTSL4, and NELL2) that could evaluate the prognosis of patients with ESCC. We also found that there were significant different therapeutic responses to chemotherapy and targeted drugs between the high-risk and low-risk groups of patients defined by the signature. More interestingly, the expression of four genes and an immune function analysis suggested that this *ECM-related signature gene might play important roles in the change of* the tumour microenvironment.

## Materials and Methods

### Patients and Public Datasets

All bioinformatics data were anonymous, obtained from the GSE53625 dataset in the GEO database, and their use did not require ethical approval.

### Identification of Differentially Expressed ECM-Related Genes

The esophageal squamous cell carcinoma dataset GSE53625 was downloaded from the GEO database. This dataset includes 179 cancer tissues and 179 paracancerous tissues, as well as patients’ clinical information (including age, sex, survival time, survival status, smoking, and drinking status, etc.; Table 1). The Annotate Document file was downloaded, and the data provided by the Genecode database^1^ were used to reannotate the dataset to obtain the expression matrix by R software. The limma package in R was used to normalise the matrix (Robinson et al., 2010; Smyth, 2011). The cut-off values were set as log (|FC|) ≥ 1 and *p* < 0.05. The differentially expressed ECM-related genes in ESCC were selected, based on the online Matrisome Project database (Naba et al., 2016)^2^ in November 2019 for further analysis and model construction.

**TABLE 1:** Clinical features of 179 esophageal carcinoma patients in GSE53625.

Variables		Training cohort (*n* = 91)	Testing cohort (*n* = 88)	Entire cohort (*n* = 179)
Age(years)	≤50	8	19	27
	>50	83	69	152
Gender	Male	73	73	146
	Female	18	15	33
Vital status	Alive	36	37	73
	Dead	55	51	106
Histologic Grade	Poorly	27	22	49
	Moderately	48	49	97
	Well	15	17	32
Tumour.loation	Lower	30	32	62
	Middle	51	46	97
	Upper	10	10	20
T	I–II	22	17	39
	III–IV	69	71	140
N	0	43	40	83
	1–3	48	48	96
Stage	I	5	5	10
	II	40	37	77
	III	46	46	92
Alcohol.use	YES	53	53	106
	NO	38	35	73
Alcohol.use	YES	56	58	114
	NO	35	30	65
Arrhythmia	YES	22	21	43
	NO	69	67	136
Pneumonia	YES	6	9	15
	NO	85	79	164

### GO and KEGG Analyses

Using the clusterProfiler package in R software, GO and KEGG pathway enrichment were conducted for differentially expressed ECM-related genes (Ashburner et al., 2000; Ogata et al., 2000; Yu et al., 2012). *P*-values < 0.05 were regarded to be statistically significant.

### Constructing an ECM-Related Gene Signature

A total of 179 ESCC patients with complete clinical features were randomly separated into two sets. The training cohort contained 91 samples, and the testing cohort contained 88 samples. The training cohort was designated to identify prognostic signatures and to establish prognostic risk models, while the testing cohort and the combined cohort were designated to validate the prognostic value of the models.

In the training cohort, we utilised Cox regression analysis to explore the association between differentially expressed ECM-related genes and ESCC patients’ overall survival (OS). Using univariate Cox regression, these genes were considered to be necessary when the *P*-value was < 0.05. We used Lasso regression to filter the numbers of genes for ESCC patients. Finally, we applied the multivariate Cox regression analysis to estimate the significance of each gene as an independent prognostic factor for patient OS. Based on the linear regression coefficients originating from the multivariate Cox regression analysis in each cohort, a prognostic risk model was established in the training cohort. This signature consisted of the expression level of four ECM-related genes (CST1, NELL2, ADAMTSL4, and ANGPTL7) and were weighted by the estimated regression coefficients of the multivariate Cox regression analysis.

Based on the median value referring to the risk score results, we classified 91 ESCC patients with prognostic information into high-risk and low-risk groups. The Kaplan-Meier (K-M) patient survival analysis was then plotted separately for the two groups.

### Evaluation and Verification of ECM-Related Gene Signature

After constructing the risk model, we applied it to the testing and entire cohort to validate the accuracy of the model. The receiver operating characteristic curve (ROC) was also determined to assess the predictive power of the models in the training cohort, testing cohort, and the entire cohort, respectively.

### Gene Set Enrichment Analysis

We unitised gene set enrichment analysis (GSEA) (Kuleshov et al., 2019; Reimand et al., 2019) to investigate the possible mechanisms and functions of the high-risk and low-risk groups, including Gene Ontology (GO) and Kyoto Encyclopedia of Genes and Genomes (KEGG) functional analyses. The number of random sample permutations was set at 1,000, and the significance threshold was set at *p* < 0.05.

### Estimation of TME and Tumour-Infiltrating Immune Cells

Stromal, immune cells and ESTIMATE scores of TME were determined by the “ESTIMATE” R package (Yoshihara et al., 2013). The relationships between signature genes and immune cells were determined by the TIMER database^3^ (Li et al., 2017a).

### Drug Therapeutic Response Prediction

We used the R package “pRRophetic” to estimate the drug therapeutic response that was determined by the half maximal inhibitory concentration (IC50) of each ESCC patient on the Genomics of Drug Sensitivity in Cancer (GDSC) website (Garnett et al., 2012; Yang et al., 2012; Iorio et al., 2016).

### Independent Predictive Factor Assessment

Combined with the clinical features (including age, sex, tobacco use, alcohol use, tumour location, tumour grade, pathologic stage, arrhythmia, and pneumonia) of patients with ESCC, univariate Cox regression analysis was performed with these features as independent variables and total survival time as the dependent variable. The multivariate regression analysis was performed with the univariate analysis results of significant *p*-values (*p* < 0.05). We calculated the hazard ratio (HR), 95% confidence interval, and two-sided *P*-values.

### Cell Lines, Cell Culture, and Western Blotting

The human ESCC cell lines Eca109 and KYSE150 and human normal esophageal squamous epithelial cells (HET-1A) were cultured in RPMI-1640 supplemented with 10% foetal bovine serum and 1% penicillin–streptomycin or Dulbecco’s modified Eagle’s medium (high-glucose medium) and minimal essential medium buffer in a humidified atmosphere of 5% CO_2_ maintained at 37°C.

Equal amounts of protein were loaded and separated using sodium dodecyl sulfate-polyacrylamide gel electrophoresis (SDS-PAGE). After transfer to a polyvinylidene difluoride membrane, the proteins were blocked with 5% bovine serum albumin (BSA) and incubated at 4°C overnight with the following primary antibodies: CST1 (Proteintech, China, 1:800), NELL2 (Proteintech, China, 1:800), ADAMTSL4 (Proteintech, China, 1:800), and ANGPTL7 (Proteintech, China, 1:800). Subsequently, the samples were incubated with horseradish peroxidase (HRP)-conjugated secondary antibody for 2 h. The targeted proteins were detected and visualised with an enhanced chemiluminescence system (GE Healthcare) and X-ray film (GE Healthcare). Beta-actin was used as a loading control.

### Immunohistochemistry (IHC)

For IHC, tissue microarray chips containing 66 pairs of ESCC and adjacent tissues were obtained from the First Affiliated Hospital of Shihezi University in Xinjiang. Immunohistochemistry (IHC) staining was performed as previously described (Liu et al., 2019). These microarray chips were sequentially incubated with rabbit antibodies against CST1 (Proteintech, China, 1:400), NELL2 (Proteintech, China, 1:400), ADAMTSL4 (Proteintech, China, 1:400), and ANGPTL7 (Proteintech, China, 1:400). HRP-labeled secondary antibody was added the next day. Afterward, the nuclei were counterstained with haematoxylin when adding the DAB solution. Analysis of the staining was independently assessed by two experienced pathologists based on the percentage of positive cells and the staining density. The final score was equal to the staining intensity score, multiplied by the average percentage of positive cells.

### Statistical Analyses

Statistical analyses were performed with R software (Version 3.6.1). Univariate, lasso, and multivariate Cox regression models were utilised to evaluate the prognostic value. The Kaplan-Meier (KM) survival analysis, followed by a log-rank testing, was applied to analyse the overall survival (OS) time of the risk groups. We then confirmed the accuracy of this model in the test and the entire data set. Finally, hazard ratios (HRs) and 95% confidence intervals (CIs) were calculated to describe the relative risk. A *P*-value < 0.05 was regarded as a statistically significant difference.

## Results

### Identification of ECM-Related Genes in ESCC

**FIGURE 1:**
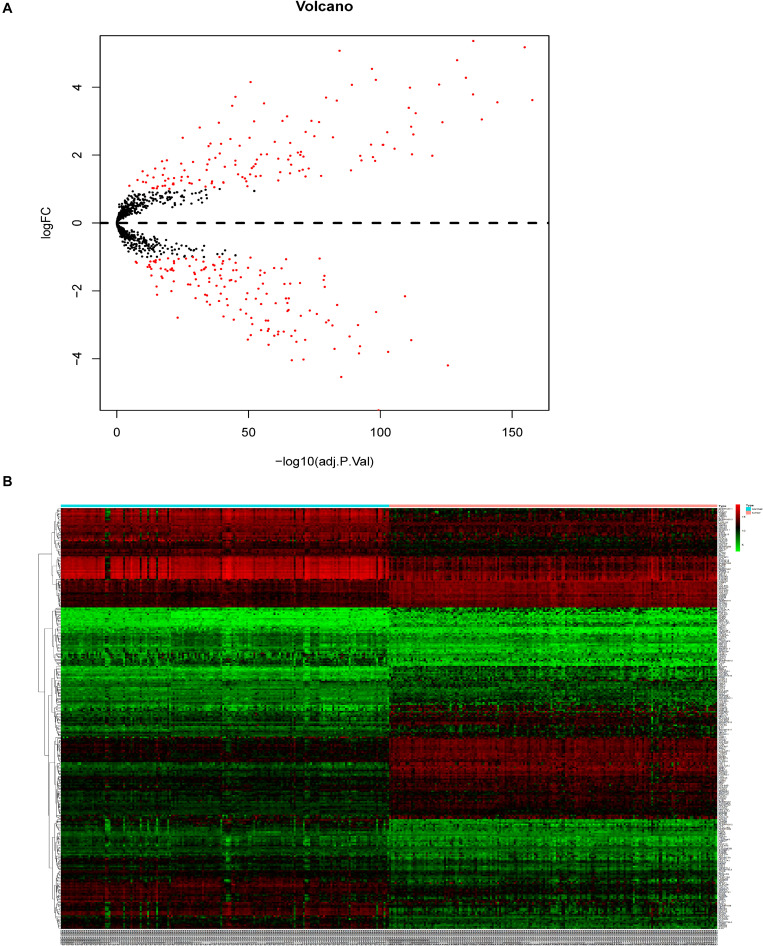
Identification of DEGs in patients with ESCC. **(A)** Volcano plot of identified DEGs. **(B)** Heatmap.

A total of 3,481 differentially expressed genes (DEGs) (log2|fold change| >1.5 and adj. *P* < 0.05) were identified by the expression levels of lncRNAs and mRNAs between the 179 ESCC and adjacent tissues, including 1447 significantly overexpressed and 2034 significantly downregulated genes (Supplementary Figure 1A). From the 3481 DEGs in ESCC, we selected 280 ECM-related genes present in the Matrisome Database (logFC > 1 or logFC < **−**1, adjusted *P*-value < 0.05) (Figure 1B).

**FIGURE 2:**
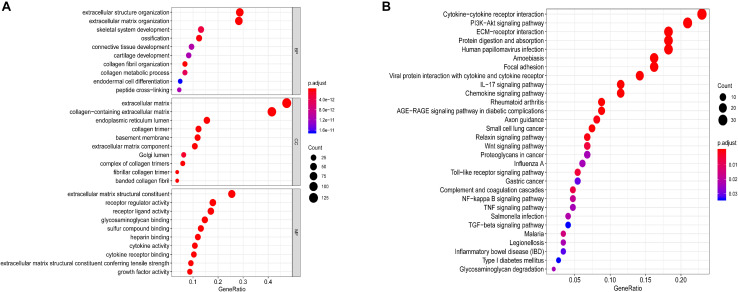
Functional annotations and analysis. **(A)** GO analysis and **(B)** KEGG analysis of differentially expressed ECM-related genes.

GO and KEGG pathway enrichment analyses of 280 ECM-related genes were conducted (adj. *P* < 0.05). As shown in Figure 2A, the top 10 GO analysis revealed that functional categories were enriched in the extracellular structure organisation and extracellular matrix organisation in BP; extracellular matrix and collagen-containing extracellular matrix in CC; and the extracellular matrix structural constituent and cytokine-cytokine receptor interaction in MF (adj. *P* < 0.05). KEGG functional analysis revealed that the top 30 pathway function categories (adj. *P* < 0.05) were significantly enriched in the ECM-receptor, cytokine-cytokine receptor interaction, and Wnt signalling pathways (Figure 2B). These results suggest that the ECM genes might affect the prognosis of patients with ESCC through the biological processes and pathways above.

### Establishment of ECM-Related Signature in Training Cohort

**FIGURE 3:**
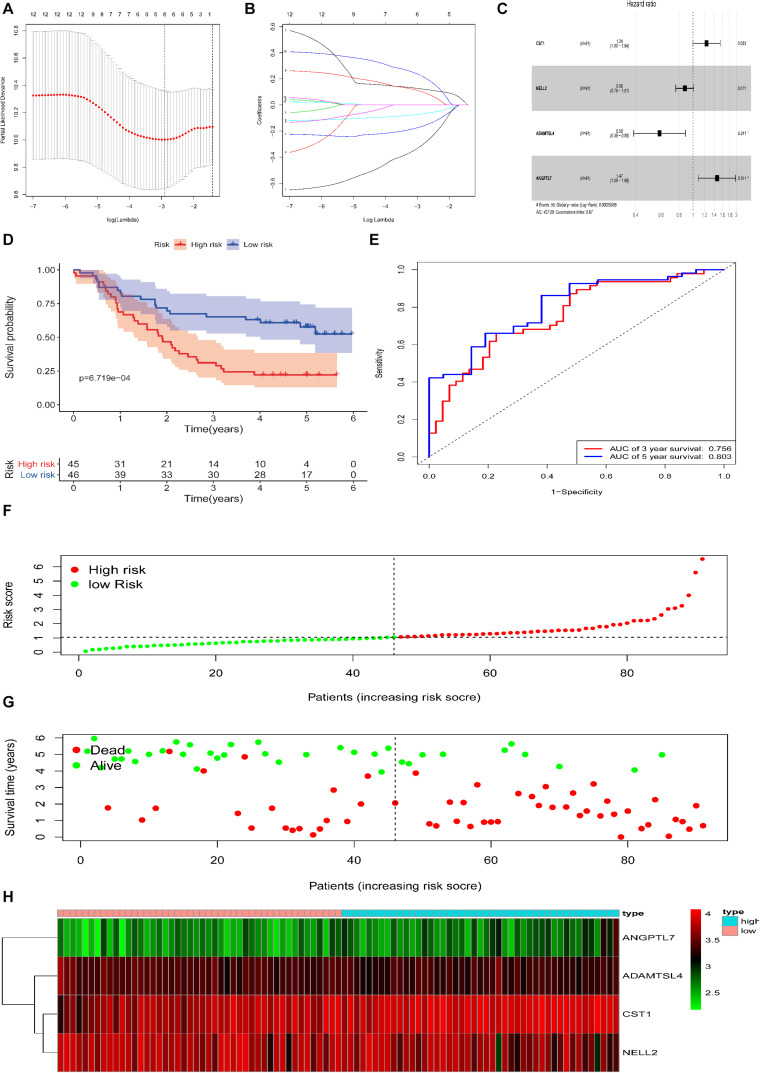
Construction of the DEG-based prognostic signature in the training cohort of patients with ESCC. **(A,B)** Lasso analysis yielded six genes for further analysis. **(C)** Multivariate regression analysis identified four genes to be included in the ECM-related prediction model. **(D)** Kaplan-Meier survival curves of the relative overall survival of the high- and low-risk groups. **(E)** ROC curve analysis of the prognostic signature. **(F)** Distribution of the four gene-based risk scores. **(G)** Vital statuses of patients in the high- and low-risk groups. **(H)** Heatmap of four-gene expression profiles in the high- and low-risk groups.

Based on the 280 DEGs, 12 genes (*P*-value < 0.05) were screened out as prognostic markers after univariate Cox analysis (Table 2). Six genes were then selected to establish a signature, due to the coefficient caused by the lasso model (Figures 3A,B). Moreover, four genes (CST1, NELL2, ADAMTSL4, and ANGPTL7, Supplementary Figure 1) were found to independently interact with the patient prognosis in the multivariate Cox regression (Figure 3C and Table 3).

**TABLE 2:** The univariate Cox analysis.

id	HR	HR.95L	HR.95H0	*p*-value
CST1	1.332712	1.091342	1.627466	0.004842
CST2	1.278036	1.063243	1.536219	0.00897
IL18	0.696589	0.524915	0.92441	0.012266
ADAMTSL4	0.640664	0.435687	0.942075	0.023618
NELL2	0.864257	0.756576	0.987265	0.031655
ADAMTS8	1.198456	1.015525	1.41434	0.032172
CLEC18B	0.76654	0.599247	0.980537	0.034308
ANGPTL7	1.368752	1.023105	1.831174	0.034534
SCUBE1	1.448719	1.025875	2.045851	0.035288
SLPI	0.85711	0.740934	0.991503	0.038006
CSTB	0.718285	0.524464	0.983736	0.039192
ADAMTSL3	1.247108	1.007082	1.54434	0.042901

**TABLE 3:** The ECM gene model information of ESCC.

id	Coef	HR	HR.95L	HR.95H	*p*-value
CST1	0.215106	1.239993	0.996972	1.542252	0.053271
NELL2	–0.13126	0.876987	0.760488	1.011333	0.071075
ADAMTSL4	–0.53464	0.58588	0.388086	0.884483	0.010957
ANGPTL7	0.384582	1.468999	1.090252	1.979321	0.011473

Based on the Cox proportional hazards regression model, the formula was as follows: risk score = (0.215106 × expression_CST__1_) + (−0.13126 × expression_NELL__2__)_ + (−0.53464 × expression_ADAMTSL__4_) + (0.384582 × expression_ANGPTL__7_).

According to the model, ESCC patients were divided into a high-risk group (*n* = 45) and a low-risk group (*n* = 46) with the median risk score in the training cohort as the cut-off. Patient OS was significantly shorter in the high-risk group than in the low-risk group (*P* = 6.719e−04, log-rank test, Figure 3D).

The area under the ROC curve analysis showed that this signature effectively predicted ESCC patient 3 and 5 year OS (AUC = 0.756 and 0.803, Figure 3E). The distribution of the four gene-based risk scores, vital statuses of patients sorted by risk score, and the four-gene expression heatmap are displayed (Figures 3F–H). Among the four genes in the signature, the coefficients of NELL2 and ADAMTSL4 were negative, suggesting that they may have a survival promotion significance, while the other genes seemed to be risk factors and were more highly expressed in the high-risk group than in the low-risk group within the training cohort.

### Validation of the ECM-Related Signature in Testing and Entire Cohort

**FIGURE 4:**
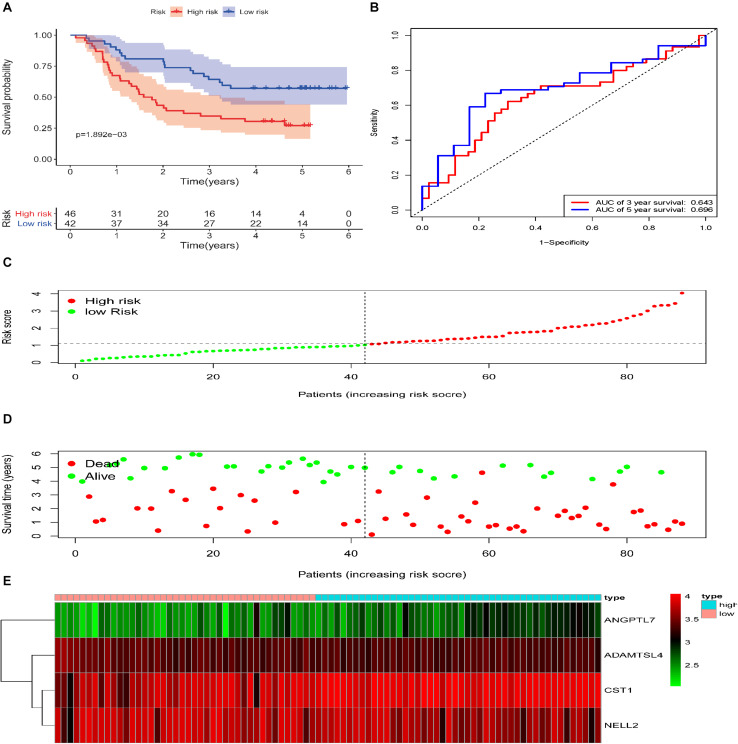
Validation of the ECM–related signature in the testing cohort of patients with ESCC. **(A)** Kaplan-Meier survival curves of the relative overall survival of high- and low-risk patients. **(B)** ROC curve analysis of the prognostic signature. **(C)** Distribution of the four gene-based risk scores. **(D)** Vital statuses of patients in the high- and low-risk groups. **(E)** Heatmap of four-gene expression profiles in the high- and low-risk groups.

**FIGURE 5:**
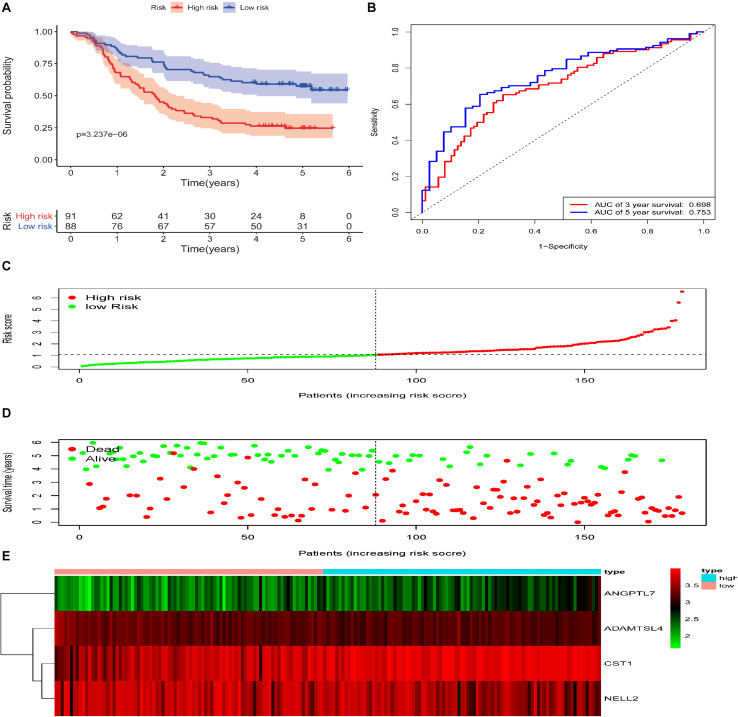
Validation of the ECM–related signature in the entire cohort of patients with ESCC. **(A)** Kaplan-Meier survival curves of the relative overall survival of high- and low-risk patients. **(B)** ROC curve analysis of the prognostic signature. **(C)** Distribution of the four gene-based risk scores. **(D)** Vital statuses of patients in the high- and low-risk groups. **(E)** Heatmap of four-gene expression profiles in the high- and low-risk groups.

To reveal the accuracy of the model, the signature model was validated in the test set and entire dataset. We further computed the risk score of ESCC patients in the test group (*n* = 88) according to the formula. The dataset was also divided into two groups using the optimal cut-off point (high-risk group vs. low-risk group, 46 vs. 42). The Kaplan-Meier curve results also suggested a significant difference in survival time between high-risk patients and low-risk patients (*P* = 1.892e−03; log-rank, Figure 4A). The time-dependent ROC analyses found that this four-gene signature-based model was an effective tool to predict ESCC patient 3 and 5 year OS, respectively (AUC = 0.643 and 0.649, Figure 4B). In addition, the Kaplan-Meier curves showed a significant difference in prognosis between the two groups (*n* = 91 vs. 88, *P* = 3.237e−06, log–rank test, Figure 5A), with a 3 and 5 year OS, respectively (AUC = 0.698 and 0.753) in the entire dataset (*n* = 179, Figure 5B). The distribution of the four gene-based risk scores and vital statuses of patients, sorted by the risk score and the four-gene expression heatmap, were also consistent with these findings (Figures 4C–E, 5C–E).

### The ECM-Related Signature as an Independent Prognostic Factor

**FIGURE 6:**
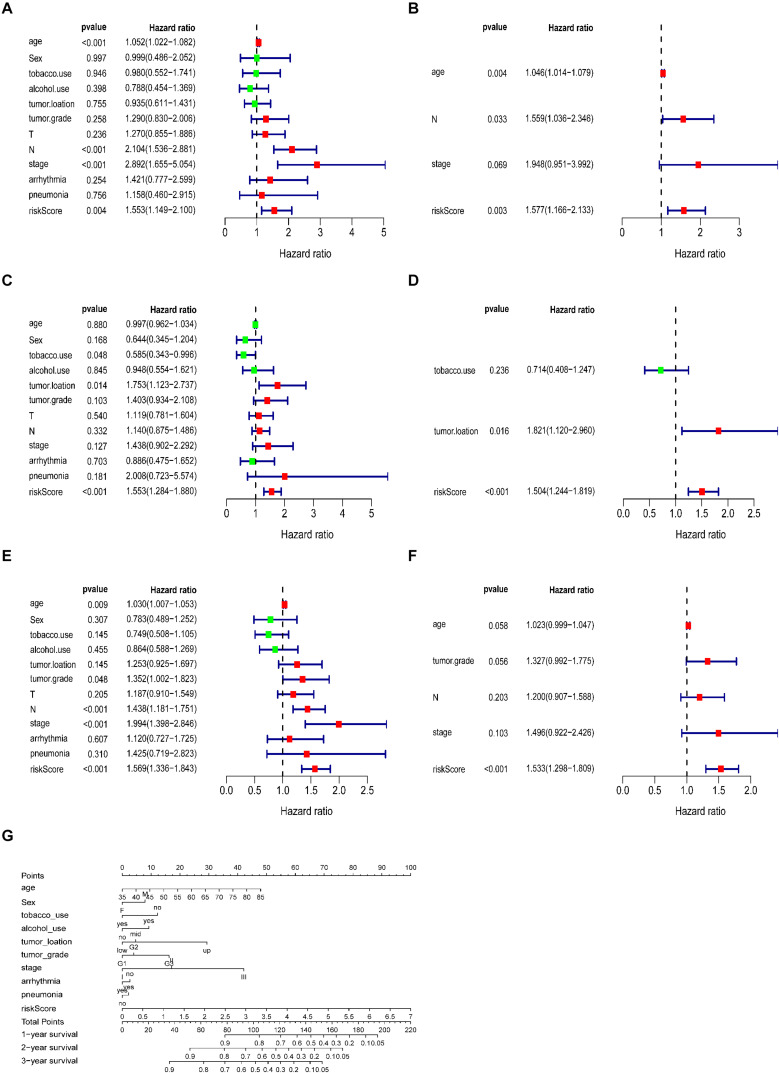
Correlation between the prognostic signature and clinical characteristics of patients with ESCC. Univariate and multivariate Cox regression analyses of correlations between the ECM-related signature and clinical characteristics and overall survival in the training **(A,B)**, testing **(C,D)** and entire cohorts **(E,F)**. **(G)** Nomogram for predicting 1-, 3-, and 5-years overall survival in patients with ESCC.

A total of 179 ESCC patients with clinical information, including sex, age, tumour grade, TNM stage, arrhythmia, pneumonia, alcohol, tobacco, and risk score, were included in further analysis. The univariate and multivariate analyses indicated that the risk score calculated from the ECM-related signature was an independent prognostic factor. (training cohort: hazard ratio = 1.557, 95% confidence interval [CI] = 1.166–2.133, *P* < 0.01; testing cohort: hazard ratio = 1.504, 95% confidence interval [CI] = 1.244–1.819, *P* < 0.001; entire cohort: hazard ratio = 1.533, 95% confidence interval [CI] = 1.298–1.809, *P* < 0.001, respectively, Figures 6A–F).

Referring to the above findings, we constructed a nomogram combining the ECM-related signature and clinical features (age, sex, stage, tumour grade, tumour location, arrhythmia, pneumonia, alcohol and tobacco use, and risk score) to directly predict 1, 2, and 3 year survival (Figure 6G).

### Therapeutic Responses for ESCC

**FIGURE 7:**
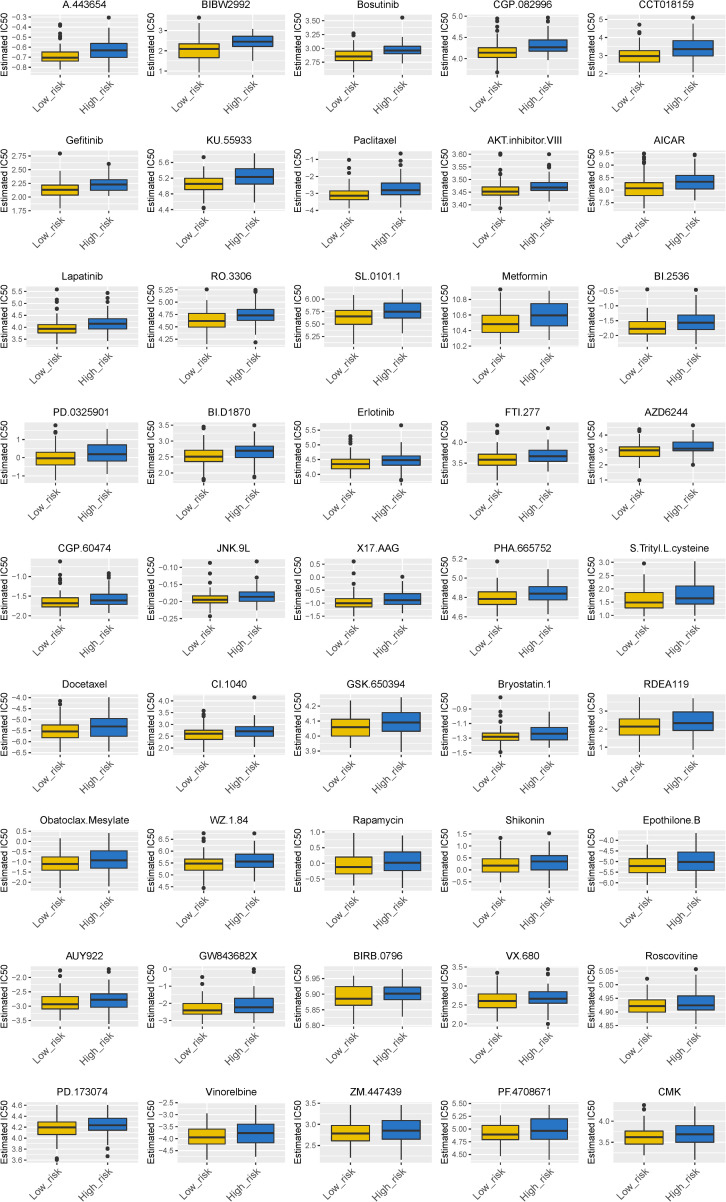
Different chemotherapeutic responses in high- and low-risk patients with ESCC. (*P* < 0.05).

We found that 24 chemotherapy and targeted drugs had great differences in estimated IC50 between high-risk and low-risk groups; specifically, patients in the high-risk group had higher IC50 values (*P* < 0.05; Figure 7). Together, these results substantiated that the signature may play a significant role in the prediction of the drug therapeutic response.

### Expression of Four Genes in ESCC Tissue and ESCC Cell Lines

**FIGURE 8:**
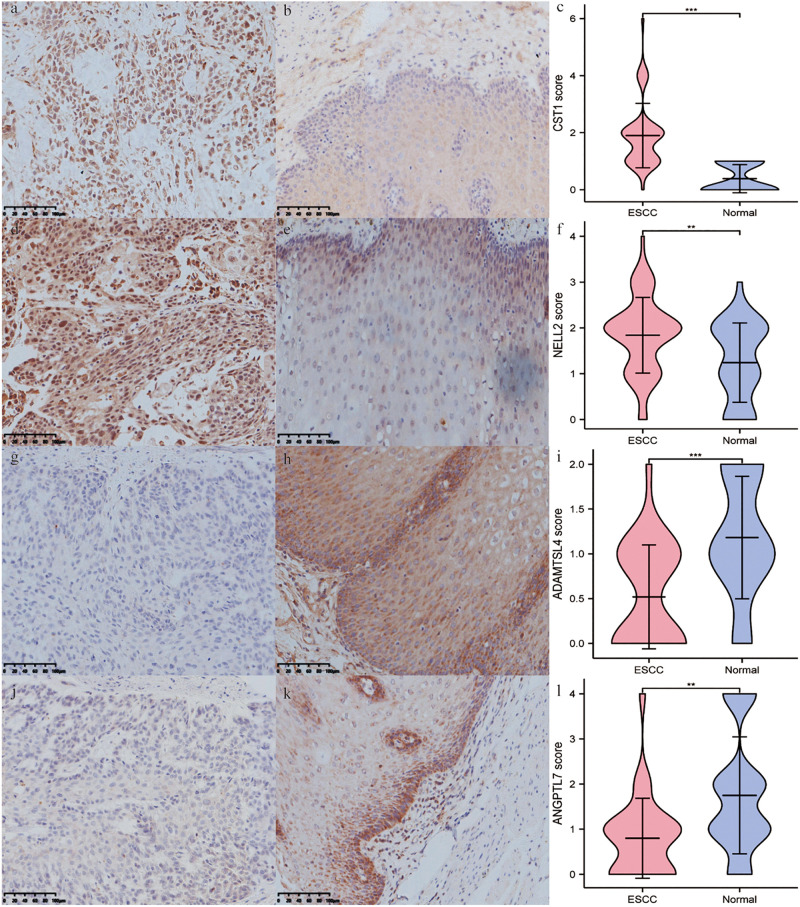
The differential expression of four ECM-related genes in ESCC tissues and adjacent non-tumour tissues detected by immunohistochemistry. The expression levels of CST1 **(a–c)** and NELL2 **(d–f)** were higher in ESCC tissues than in adjacent non-tumour tissues. In contrast, the expression levels of ADAMTSL4 **(g–i)** and ANGPTL7 **(j–l)** were lower in ESCC tissues **(e,g)** than in adjacent non-tumour tissues.

**FIGURE 9:**
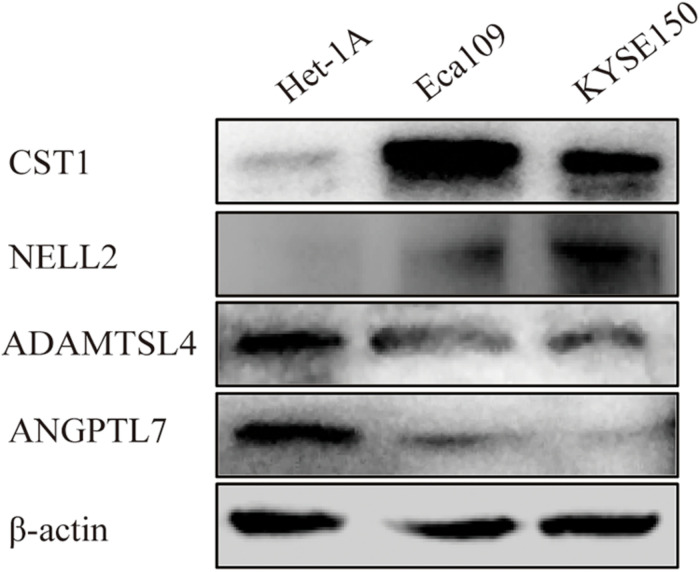
Different expression levels of CST1, NELL2, ADAMTSL4, and ANGPTL7 proteins in the ESCC cell lines Eca109 and KYSE150 and the normal cell line Het-1A detected by western blotting.

Immunohistochemistry showed that CST1, ADAMTSL4, and ANGPTL7 were mainly localised to membranes and cytoplasm, while NELL2 was primarily expressed in the nuclei in ESCC tissues. CST1 and NELL2 proteins were significantly overexpressed in tumour tissues compared with adjacent non-tumour tissues (Figures 8a–f). ADAMTSL4 and ANGPTL7 proteins were significantly overexpressed in adjacent non-tumour tissues compared with tumour tissues (Figures 8g–l). Western blot analysis revealed that the expression of CST1 and NELL2 was significantly upregulated in esophageal cancer cell lines (Eca109, KYSE150) compared with normal esophageal squamous epithelial cell lines (Het-1A), while ADAMTSL4 and ANGPTL7 was significantly increased in the Het-1A cell line and expressed at significantly low levels in Eca109 and KYSE150 (Figure 9).

### Immune Function Analysis of ECM-Related Signature Genes

**FIGURE 10:**
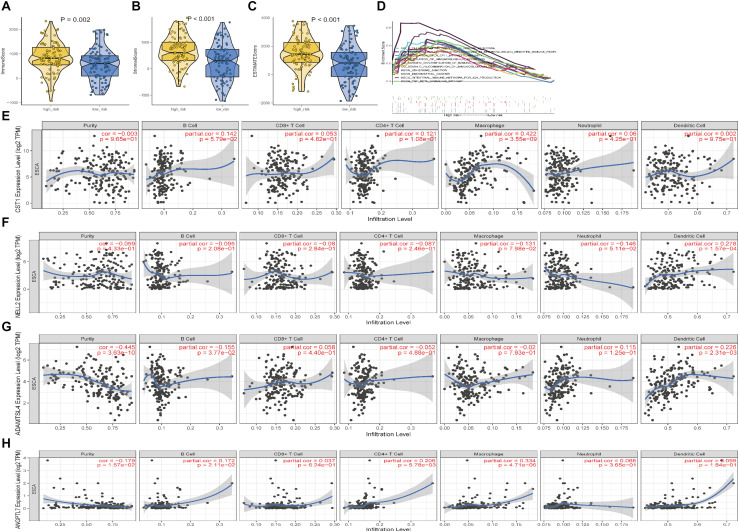
Characterisation of the TME and tumour-infiltrating immune cells in ESCC. **(A–C)** Immune, Stromal scores and ESTIMATE scores of the high- and low-risk groups in ESCC. **(D)** Gene set enrichment analysis between the high-risk and low-risk groups. **(E–H)** The correlation between the expression levels of the four genes and immune cell infiltration in TIMER.

There were significant differences in stromal and immune scores and ESTIMATE scores between the high- and low-risk groups (Figures 10A–C). After identifying the immunotherapy value of the signature, we also found a significant correlation between the expression level of four genes and the immune cell infiltration level for ESCC (Figures 10E–H). CST1 and ANGPTL7 expression was positively associated with macrophages (correlation = 0.4222 and 0.334, *p*-value < 0.01) and B cells (correlation = 0.142 and 0.172, *p*-value < 0.01), while ADAMTSL4 and NELL2 expression was negatively related to tumour purity and macrophages, respectively (*p*-value < 0.01). GSEA enrichment analysis also showed that the high-risk group defined based on the signature was obviously enriched in B cell activation, regulation of immunoglobulin, and other immune-derived factors (*P* < 0.05; Figure 10D). *These results revealed that the ECM-related signature genes may play significant roles in the changes of TME.*

## Discussion

ESCC remains one of the most common aggressive malignancies and the sixth leading cause of cancer-associated mortality in China (He et al., 2014; Su et al., 2019). Despite the development of new diagnoses and treatment approaches for ESCC in recent decades, there has been only a slight reduction in its incidence and mortality worldwide. The ECM, which can be altered in the occurrence and progression of tumours, has attracted extensive attention due to its relationship with immune processes, and several ESCC studies based on the ECM have been reported (Erdogan and Webb, 2017; Giussani et al., 2018; Eble and Niland, 2019). However, only a few biomarkers with sensitivity and specificity are available to evaluate the prognosis and therapeutic responses of patients with ESCC.

In the present study, for the first time, we identified and verified a novel ECM-related signature (consisting of ANGPTL7, ADAMTSL4, CST1, and NELL2) that effectively predicts ESCC patient overall survival. In the training cohort, patients in the high-risk group had a shorter survival time than those in the low-risk group. The ROC curve presented a relatively high prognostic prediction value, with an AUC of 0.756–0.803. Moreover, the multivariate Cox regression model indicated that this signature acted as an independent risk factor after adjustment for several clinical features, such as age, tumour grade, tumour size, and lymph node status. When patients were stratified based on pathological clinical features, the signature remained a robust prognostic tool. Similar results were observed with the validation of the model in the testing set and the entire pool of patients. Importantly, we also found that 24 chemotherapy and targeted drugs had great differences in estimated IC50 between the high-risk and low-risk groups, established based on our immune-related model, further indicating that this signature could play a pivotal role in the evaluation of prognosis and therapeutic responses for ESCC patients.

In addition, the functional and pathway enrichment analysis showed the potential functions of these four genes and identified several important biological processes and pathways associated with human cancers. GO analysis showed that the DEGs were enriched in functional categories such as receptor regulator activity and cytokine receptor binding, while KEGG functional analysis revealed that the top 30 significantly enriched pathway functional categories (adj. *P* < 0.05) included the IL-17 signalling pathway, ECM-receptor interaction, and cytokine-cytokine receptor interaction. GSEA enrichment analysis also confirmed that the high-risk group, defined based on the signature, was obviously enriched in B cell activation, regulation of immunoglobulin, and other immune-derived functions (*P* < 0.05), suggesting that the signature-associated genes might affect ESCC patients with poor prognosis through the immune processes and pathways above.

A further study showed that the four genes in the signature were closely correlated with immune score and stromal score. In the literature, stromal and immune cells have indispensable roles in the TME of multiple cancers (Yoshihara et al., 2013; Liu et al., 2018). These results indicated that this signature may play a vitally significant role in tumour progression through the immune system. Interestingly, after comparing the infiltration of seven immune cell types with the expression of identified genes, we further demonstrated the effect of TME infiltration of immune cells on the prognosis of ESCC patients. ANGPTL7 belongs to a family of secreted angiopoietin-like proteins with reported functions in the regulation of angiogenesis (Carbone et al., 2018), cancer migration, and invasion (Kuo et al., 2013). In addition, this gene promotes proinflammatory responses in macrophages by modulating the P38 MAPK signalling pathway (Qian et al., 2016). Previous studies have shown that tumour-associated macrophages play an important role in the progression and metastasis of ESCC (Wang et al., 2017; Lu et al., 2019). For instance, X. Jia’s group found that CCL2 in ESCC prompts the recruitment of tumour-associated macrophages and induces immune escape through PD-1 signalling (Yang et al., 2020). These results, along with our findings, indicate that this gene had a positive effect on macrophages, CD8 + T cells, and CD4 + T cells, and the negative relationship with tumour purity. ADAMTSL4, as a secreted glycoprotein, is a novel human biomarker in the regulation of immune-associated treatment, participating in microfibril formation and function (Hubmacher and Apte, 2015), and it is closely linked to immune-related biological processes in glioblastoma multiform (GBM) (Hubmacher and Apte, 2015). Consistently, ADAMTSL4 showed a similar behaviour in our study, suggesting that it has an inverse interaction with tumour purity. We therefore hypothesise that ANGPTL7 and ADAMTSL4 might be mainly involved in regulating the formation and progression of ESCC by the above immune processes. Cysteine protease is a proteolytic enzyme that is widely distributed in tissues and has many functions, including the degradation of the extracellular matrix and the regulation of immune responses such as inflammation (Lah et al., 1993). The upregulation of CST1 expression is closely associated with poor prognosis in breast cancer patients (Dai et al., 2017) and colon cancer (Li et al., 2017b; Jiang et al., 2018), consistent with our results verifying the expression of CST1 in esophageal cancer tissues and cell lines by immunohistochemistry (IHC) and western blotting. Although recent studies have also shown that CST1 is a key antigen in humoral immune regulation (Deshmukh et al., 2020), there are very few studies on the role of CST1 in the tumour microenvironment. Neural epidermal growth factor-like-like 2 (NELL2) has been reported to serve important functions in the development, survival, and activity of neurons in animals (Jaworski et al., 2015). Some research has confirmed that Nell 2 can inhibit the migration of renal carcinoma cells (Nakamura et al., 2015). This is consistent with our IHC and western blotting findings that NELL is a protective factor for esophageal squamous cell carcinoma. A recent study, published in the journal Science, showed that Nell 2 is closely related to male fertility (Kiyozumi et al., 2020). There are very few reports on the relationship between NELL2 and the tumour microenvironment, so it is necessary to study the mechanism by which NELL2 regulates the tumour microenvironment.

The above results show that poor prognosis in ESCC patients may mainly be attributed to lower immune activity and inhibited immune reactivity in the TME, which can help to overcome immune suppression and enhanced antitumour immunity. According to these changes, high-risk patients with ESCC may benefit from immunotherapy and chemotherapy. Nevertheless, there are some limitations that should be noted. First, only limited data were used for the construction of signatures, and more robust specimens should be collected in the future. Next, the biological functions of the four identified genes, especially their association with immune infiltration, should be further assayed.

In summary, for the first time, we established an immune-related signature based on ECM genes that serves as an independent prognostic factor for ESCC patients and reflects the intensity of the immune response in the ESCC microenvironment. The signature showed promising sensitivity and specificity for the prediction of survival and chemotherapy responses, thereby providing new prognostic and treatment strategies for this fatal disease.

## Data Availability Statement

Publicly available datasets were analysed in this study. This data can be found here: https://www.ncbi.nlm.nih.gov/geo (GSE53625).

## Author Contributions

HZ, QS, and ZY designed the experiments and wrote the manuscript, contributed equally to this work. KW, ZZ, and ZH collected data. XC and FL conducted the experiments. All authors contributed to the article and approved the submitted version.

## Conflict of Interest

The authors declare that the research was conducted in the absence of any commercial or financial relationships that could be construed as a potential conflict of interest.

## Footnotes

1 https://www.gencodegenes.org

2 http://matrisomeproject.mit.edu/other-resources/human-matrisome/

3 https://cistrome.shinyapps.io/timer/

## Supplementary Material

Supplementary Figure 1 The prognostic values of four genes in patients with ESCC. **(A)** CST1, **(B)** NELL2, **(C)** ADAMTSL4, and **(D)** ANGPTL7.
