# Supplementary figures and images for: An Extracellular Matrix-Based Signature Associated With Immune Microenvironment Predicts the Prognosis and Therapeutic Responses of Patients With Oesophageal Squamous Cell Carcinoma
Source: Front Mol Biosci. 2021 Mar 18;8:598427. doi: 10.3389/fmolb.2021.598427 (PMC8044946; doi:10.3389/fmolb.2021.598427)

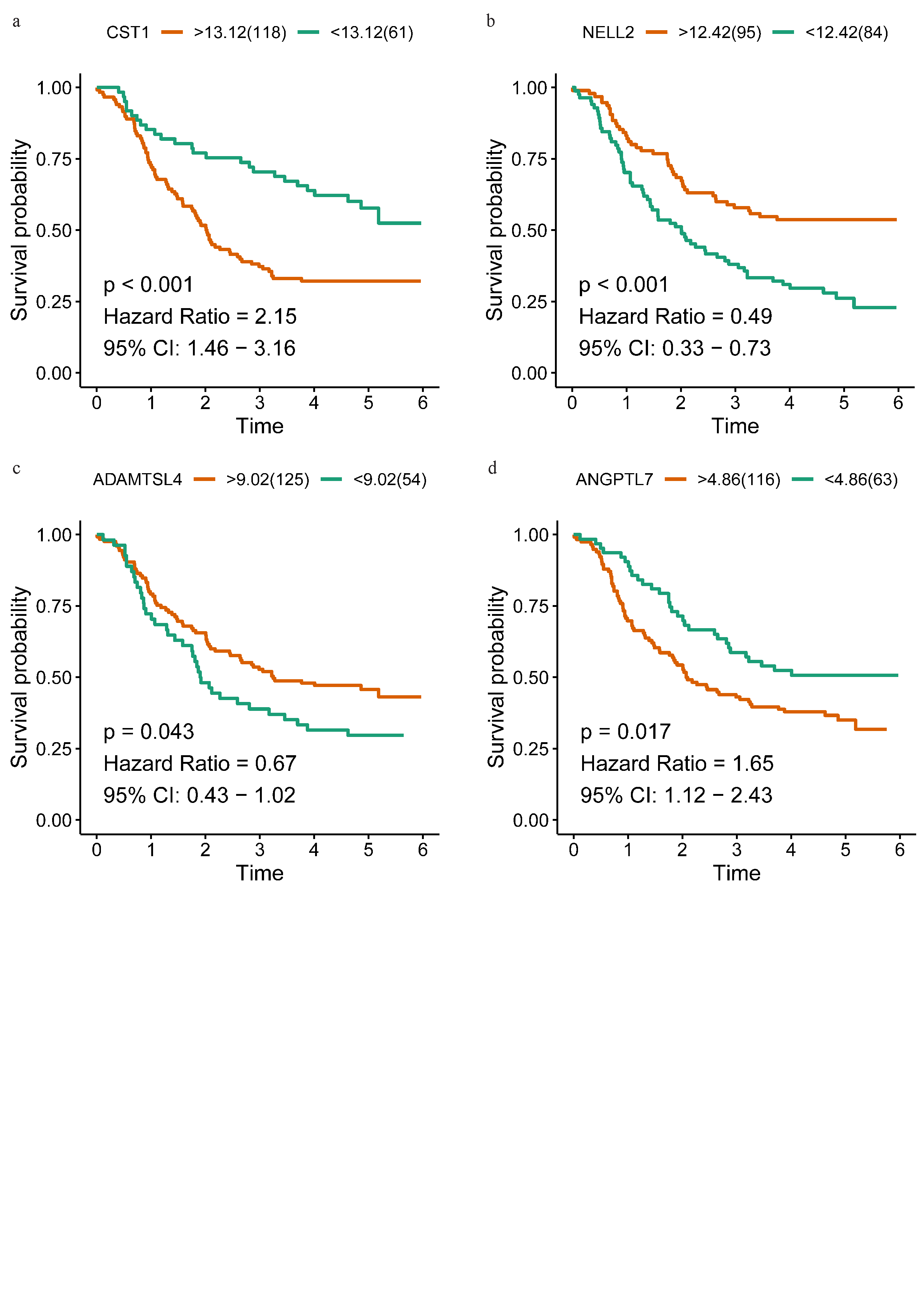

Supplement: Supplementary Figure 1 — The prognostic values of four genes in patients with ESCC. (A) CST1, (B) NELL2, (C) ADAMTSL4, and (D) ANGPTL7. [file Image_1.TIF]
